# A trauma network with centralized and local health care structures: Evaluating the effectiveness of the first certified Trauma Network of the German Society of Trauma Surgery

**DOI:** 10.1371/journal.pone.0194292

**Published:** 2018-03-14

**Authors:** Antonio Ernstberger, Michael Koller, Florian Zeman, Maximilian Kerschbaum, Franz Hilber, Eva Diepold, Julika Loss, Tanja Herbst, Michael Nerlich, Trauma Centers of the Trauma Network of Eastern Bavaria (TraumaNetzwerk Ostbayern − TNO)

**Affiliations:** 1 Department of Trauma Surgery, University Medical Center Regensburg, Regensburg, Germany; 2 Center for Clinical Studies, University Medical Center Regensburg, Regensburg, Germany; 3 Institute of Epidemiology and Preventive Medicine, University of Regensburg, Regensburg, Germany; Klinikum rechts der Isar der Technischen Universitat Munchen, GERMANY

## Abstract

**Background:**

Trauma is a global burden of disease and one of the main causes of death worldwide. Therefore, many countries around the world have implemented a wide range of different initiatives to minimize mortality rates after trauma. One of these initiatives is the bundling of treatment expertise in trauma centers and the establishment of trauma networks.

Germany has a decentralized system of trauma care medical centers. Severely injured patients ought to receive adequate treatment in both level I and level II centers. This study investigated the effectiveness of a decentralized network and the question whether level I and level II centers have comparable patient outcome.

**Materials and methods:**

In 2009, the first trauma network DGU^®^ in Germany was certified in the rural area of Eastern Bavaria. All patients admitted to the 25 participating hospitals were prospectively included in this network in the framework of a study sponsored by the German Federal Ministry of Education and Research between March 2012 and February 2014. 2 hospitals were level I centers (maximal care centers), 8 hospitals were level II centers, and 15 hospitals were level III centers. The criterion for study inclusion was an injury severity score (ISS) ≥ 16 for patients´ primarily admitted to a level I or a level II center. Exclusion criteria were transferal to another hospital within 48 h, an unknown revised injury severity classification II score (RISC II), or primary admittance to a level III center (n = 52). 875 patients were included in the study.

Univariate analyses were used regarding the preclinical and clinical parameters, the primary endpoint mortality rate, and the secondary endpoints length of stay, organ failure, and neurological outcome (GOS). The primary endpoint was additionally evaluated by means of multivariable analysis.

**Results:**

Indices for injury severity (GCS, AIS_Head_, ISS, and NISS) as well as the predicted probability of death (RISC II) were higher in level I centers than in level II centers.

No significant differences were found between the mortality rate of the unadjusted analysis [level I: 21.6% (CI: 16.5, 27.9), level II: 18.1% (CI: 14.4, 22.5), p = 0.28] and that of the adjusted analysis [level I SMR: 0.94 (CI: 0.72, 1.21), level II SMR: 1.18 (CI 0.95, 1.48) SMR: expected vs. calculated mortality rate according to RISC II]. Multivariable analysis showed a survival advantage of patients admitted to a level I center with a probability of death of 13% (RISC II). The number need to treat was 10 patients.

**Discussion:**

This study showed that a rural trauma network with centralized and local structures may achieve equivalent results with regard to mortality rates to those obtained in level I and level II centers. These results were furthered by a certain preclinical centralization (24/7 air rescue) of patients. The study also showed a survival advantage of patients admitted to a level I center with a probability of death of 13%. Preclinical and initial clinical evaluation with regard to probable mortality rates should be further improved to identify patients who would benefit from admittance to a level I center.

## Introduction

Clinical research remains focused on chronic diseases. One reason for this focus may be the ready availability of data. Clinical research and health care research into the area of emergency care are much more difficult with regard to study management and data acquisition. For this reason, research into emergency care is rather limited. This underrepresentation is in stark contrast to the enormous cost required for acute and emergency treatment to provide excellent emergency services not only in Germany but also in all other high-income countries. Above all, treatment of the most seriously injured patients should be emphasized in this respect. Serious injuries are associated with higher economic costs than cancer and cardiovascular diseases [1, 2]. Thus, the World Health Organization (WHO) has declared trauma a key target disease in the future [3–5]. The high relevance of trauma is also reflected in the total number of fatalities due to injury that amounted to over 20,000 –including 3,300 road fatalities–alone in Germany in 2013 [6].

### Improving emergency care through trauma networks

Emergency care is being permanently improved worldwide. One approach to improving emergency care in many countries has been the establishment of trauma networks. However, it is still unclear if the establishment of trauma networks has a positive effect on the emergency treatment of most seriously injured patients. International publications are contradictory [7–11]. To lastingly improve the quality of emergency treatment necessitates solid knowledge of intersectoral and interdisciplinary processes [12] that need to be executed as quickly as possible when providing emergency treatment for most seriously injured patients [13].

### Trauma network initiatives in Germany

The federal states of Germany used to differ significantly in their survival probability after road traffic accidents [14]. In 2006, urban areas with higher hospital density were statistically better than rural areas with lower hospital density. Moreover, hospitals also differed with regard to their survival probability [15]. The German Society of Trauma Surgery (DGU) published the White Paper on seriously injured patients in 2006 and the second edition in 2012 to ensure comparable outcomes on a national level by optimizing emergency care [16, 17]. The White Paper specified for the first time the structural conditions for trauma centers and recommended the merging of individual hospitals to regional trauma networks. The trauma network initiative aimed at establishing a nationwide network of hospitals in Germany to raise regional differences in the emergency treatment of most seriously injured patients to the same high level of availability and quality, thus permanently improving emergency care on a national level.

A three-tier classification of centers similar to the trauma system in the United States (US) was introduced in Germany. Hospitals have been classified as ‘local trauma centers’ similar to US level III, ‘regional trauma centers’ similar to US level II, and supraregional trauma centers similar to US level I. The structural quality of each hospital is examined by means of an independent audit that is repeated every three years. Each center is categorized into one group of the three levels of health care, and this categorization necessitates proof of a specified minimum number of most seriously injured patients. After the audit of each hospital of the trauma network, the entire network can be certified.

In contrast to the highly centralized US system [7, 8], Germany has retained its decentralized system based on the shortest possible transportation time of most seriously injured patients that had already been established before the network initiative. Thus, using the inclusive/exclusive terminology, the German system could be characterized as “over”-inclusive [18, 19].

The transportation time of a most seriously injured patient to a level I or level II center should not exceed 30 minutes. If this is not possible, the patient should be stabilized in a level III center and then be transferred to a level II or level I trauma center.

This philosophy is mirrored in the density of level I centers. In Germany, the catchment area of a level I center comprises on average 0.9 million inhabitants in comparison to 1.5 million inhabitants in the US, 2.2 million inhabitants in Canada, and 2.5 million inhabitants in the UK [www.dgu-online.de, www.facs.org, www.traumacanada.org, www.nhs.uk]. The average area covered by a level I center in Germany is 360,000 square kilometers in contrast to over 9.5 million square kilometers in the US and Canada. In Germany, the treatment outcome of most seriously injured patients in level II centers are also supposed to be comparable to the treatment outcome in level I centers.

### Process quality in the trauma network

The White Paper covers structural as well as process quality. At least 50% of surgeons working in the shock room have to be trained in advanced trauma live support (ATLS; https://www.facs.org/quality%20programs/trauma/atls). The German Society of Trauma Surgery has issued the S3 guideline on polytrauma (German S3 Guideline on Treatment of Patients with Severe and Multiple Injuries) that provides evidence-based recommendations on process quality [20].

### Aim of the study

The objective of this publication was the evaluation of the first certified trauma network of the German Society of Trauma Surgery in terms of a small area analysis of a rural network. For this analysis, level I and level II centers were compared regarding the following criteria:

1. The distribution of patients,
2. Injury patterns and injury severity level,
3. The quality of the results.

## Materials and methods

### Study design

This prospective multi-center cohort study compared level I centers with level II centers of the Trauma Network of Eastern Bavaria with regard to the treatment of most seriously injured patients (ISS≥16). The study period was 24 months. The treatment of most seriously injured patients in the study region was examined in terms of an observational study without any intervention. Data were collected anonymously and documented with the data set of the TraumaRegister^QM^ of the German Society of Trauma Surgery. The Polytrauma Health Care Quality Outcome (POLYQUALY) Study (“Outcome after major trauma in a certified trauma network: comparing standard vs. maximum care facilities”) was approved by the Ethics Committee of the University of Regensburg (number 10-101–0077) and sponsored by the German Federal Ministry of Education and Research (01GY1153). The study is registered in the data base of the German Network for Healthcare Research (VfD_Polyqualy_12_001978) and in the German Register for Clinical Studies (number DRKS00010039). The study protocol has been published elsewhere [21].

### Study region and sample of the analysis

**Fig 1:**
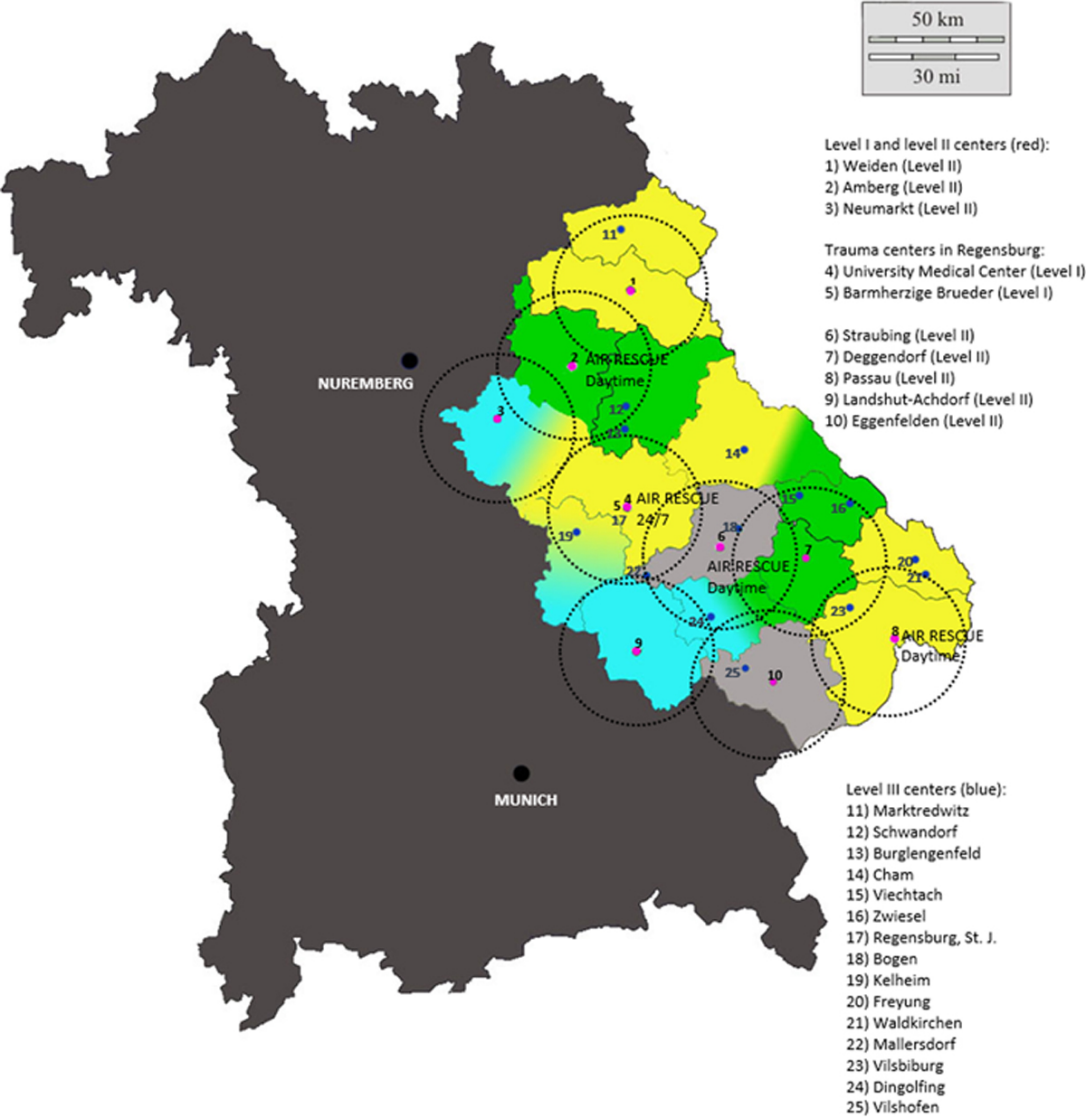
Study region. Red, Study Centers; Circles, 30 km Radius; Colored, Actual Catchment Areas of the Trauma Centers for Ambulance Service. Changed and reprinted with the permission of d-maps.com.

The study included most seriously injured patients with an ISS of ≥16 who had met with an accident in Eastern Bavaria. This mainly rural area consisting of the administrative districts of Lower Bavaria and Upper Palatinate covers an area of about 20,000 square kilometers and has approximately 2.3 million inhabitants. Eastern Bavaria also borders the Czech Republic and Austria. Overall, 25 hospitals (2 level I centers, 8 level II centers, and 15 level III centers, Fig 1) of the Trauma Network of Eastern Bavaria participated in the study.

In the study region, one dual use helicopter stationed at a level I center is available 24/7. During daylight, three more emergency rescue helicopters are available in the towns of Amberg, Straubing, and Passau/Suben. The map on Fig 1 shows the level I and level II centers of Eastern Bavaria in red, each with their respective radius of 30 kilometers that would allow ground transportation within 30 minutes. Furthermore, the actual catchment areas of the centers in the ambulance service regions are marked in color.

Data collection started on 1 March 2012 and ended after 24 months on 28 February 2014. The inclusion criteria of the primary data set were as follows: patients with an ISS of ≥16 admitted to the shock room of one of the hospitals of the Trauma Network of Eastern Bavaria between 1 March 2012 and 28 February 2014, patients requiring intensive care, and patients dying in the shock room. The primary data set also included transferred patients.

Inclusion criteria for the present analysis were:

- Primary admittance of the patient at the site of the accident (no referrals from other hospitals),
- Admittance to a level I or level II center,
- Injury severity according to the Injury Severity Score (ISS) of ≥16.

**Fig 2:**
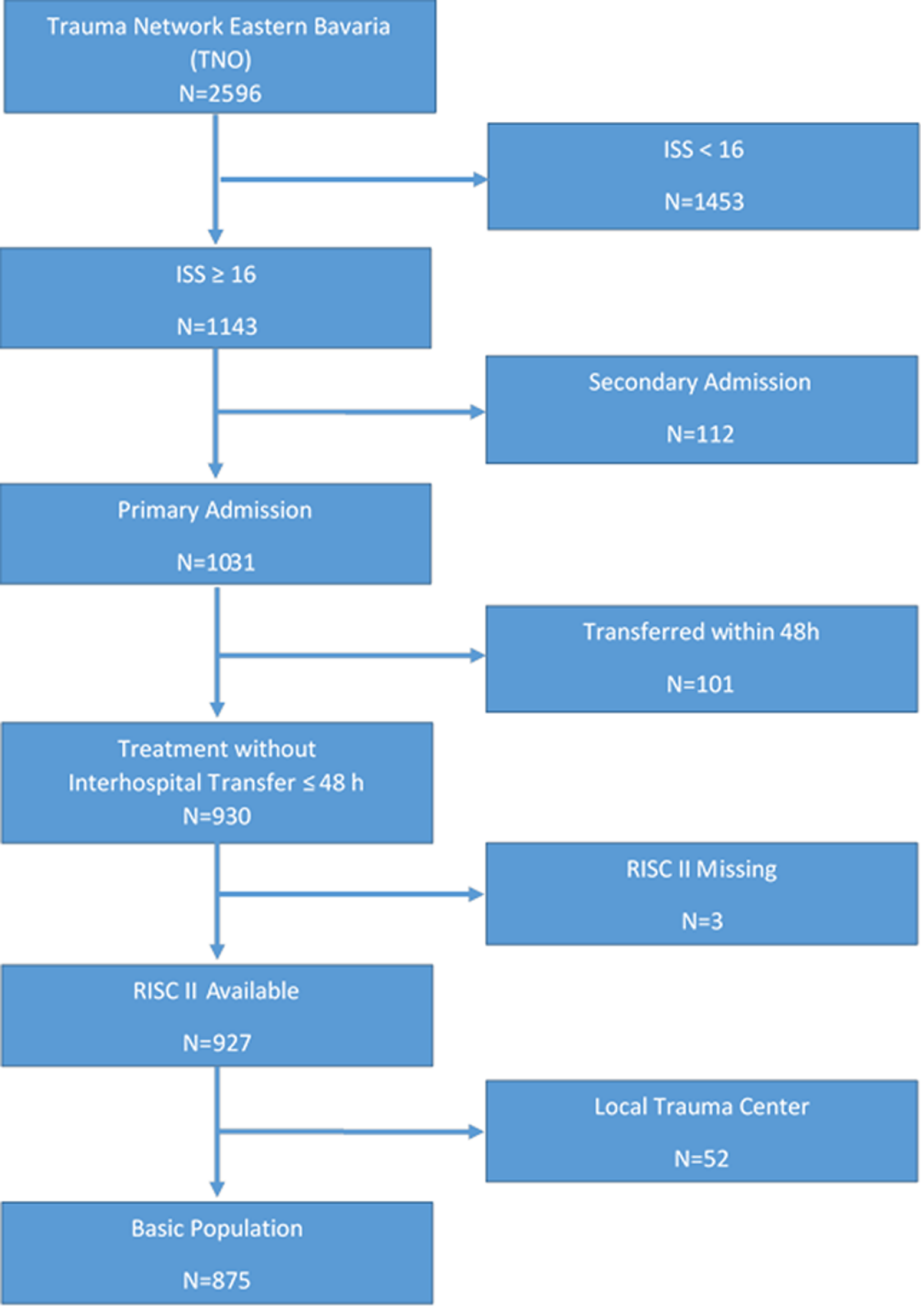
Case inclusion. Flowchart of Case Inclusion; ISS, Injury Severity Score; RISC II, Revised Injury Severity Classification II.

Exclusion criteria were unknown Revised Injury Severity Classification II (RISC II), referral to another trauma center within 48 hours, and primary admittance to a level III center (52 patients, Fig 2).

### Collection of data

The underlying set of data was taken from the TraumaRegister^QM^ DGU^®^ (http://www.traumaregister.de) that was established in 1993 and currently comprises over 600 actively participating hospitals [22–24]. The set of data is both anonymous and validated and conforms to the Utstein criteria [25, 26]. Every participating trauma center prospectively documented the demographic data of each patient admitted to the shock room, the items of the TraumaRegister^QM^ DGU^®^ regarding the cause of injury, as well as preclinical and emergency aspects such as physiological parameters and therapy. All data were initially documented in analog format. After discharge of the patient, these data were entered into the web-based TraumaRegister^QM^ DGU^®^. The further clinical course and the outcome were also recorded. If a hospital was unable to conduct the web-based recording of the data, a study assistant was provided by the study center at the maximum care hospital of the trauma network (university medical center).

The following scoring systems have been implemented in the TraumaRegister^QM^ DGU^®^ or can be calculated from its items: the Glasgow Coma Score (GCS) [27], the Abbreviated Injury Scale (AIS 2005 update 2008) [28], the Injury Severity Score (ISS) [29], the Glasgow Outcome Scale (GOS) [30], the Revised Injury Severity Classification II (RISC II) [31], and the Standardized Mortality Ratio (SMR) (Table 1). Based on the TraumaRegister^QM^ DGU^®^, the RISC II score has the highest predictive accuracy regarding survival probability of the common prognostic scoring systems [NISS (New Injury Severity Score), ISS, TRISS (Trauma and Injury Severity Score), and RISC] with an area under the curve (AUC) of receiver-operating characteristic curves (ROC) of 0.951 [31].

**Table 1:** Key scoring systems.

Scoring System	Description	Range
GCS	Glasgow Coma Scale: Assessment of neurological status at the scene of injury and in the shock room [27]	3 (poor) - 15 (excellent)
GOS	Glasgow Outcome Scale: Assessment of neurological outcome at discharge [30]	1 (death) - 5 (none/minimal deficit)
AIS	Abbreviated Injury Scale: Anatomical score to assess the severity of a single injury with regard to the mortality risk [28]	1 (minor) - 6 (maximum)
ISS	Injury Severity Score: Anatomical score to assess the severity of multiple injuries on the basis of the AIS by adding the square of the three most severely injured body regions: ((AIS_Reg1_)^2^+(AIS_Reg2_)^2^+(AIS_Reg1_)^2^) Maximum value 75 (3 x AIS 5 or by definition at least 1 x AIS 6) [29]	0–75 ISS ≥16 common edge for multiple- injured patients
RISC II	Revised Injury Severity Classification II: Description of the probable mortality rate on the basis of 14 items by means of a risk adjustment model (AUC of ROC curve 0.951) [31]	0%-100%
SMR	Standardized Mortality Ratio: In this study: Relation of observed mortality vs. calculated mortality (RISC II)	<1 better >1 worse than prediction

Scoring systems used in the study

### Measures to ensure data quality

Data quality was a major focus in this study. The data quality dimensions completeness of participants, correctness of recorded data and completeness of the individual data sets were greatly appreciated. Details are described in the published study protocol [21].

### Statistical analysis

In a first step, the observed number of patients was compared with the predicted number of patients. The predicted number of patients was calculated on assumed ground transportation to the closest level I or level II center according to the catchment area of the respective hospital: Based on the incidence of multiple trauma patients (calculated based on the study population and the number of inhabitants of Eastern Bavaria), the predicted number of most seriously injured patients per hospital was estimated according to the number of inhabitants in the catchment area of the hospital.

The following calculation was applied:

[N] n of trauma patients ISS≥16 (study data)
[I] n of inhabitants in eastern Bavaria (official statistical data)
[ICA] n of inhabitants in the defined catchment areas of each hospital (official statistical data)
[N]/[I] = [M] incidence of trauma patients ISS≥16 per 100.000 in Eastern Bavaria
[X] n of expected trauma patients ISS≥16 for each catchment area = [ICA]x[M]

The most probable primary catchment area of each trauma center was estimated by means of different assumed values: The calculation was based on a 30 kilometer radius around the respective center—as proposed by the Whitebook Medical Care of the Severely Injured [16, 17], taking into account the maximum transportation time of 30 minutes and the borders of the ambulance service regions. If 2 hospitals were located within one ambulance service region because of the overlapping of the two 30 kilometer radii, the catchment area was divided according to the location of the hospitals. Trauma centers in the border regions with the Czech Republic that were not covered by the 30 minute transportation rule were allocated to the closest level II center (Fig 1).

In a second step, level I and level II centers were compared by means of univariate analysis: With regard to demography, vital parameters, and process parameters, ordinal and metric data were described with means and standard variations and categorical data with percentage rates. Missing values were accepted in the univariate analysis–no missing values were included in the multivariable analysis. No imputation method for missing values was used. The Number of missing cases for each variable are specified in the tables. Missing values in the univariate analyses did not exceed the 10% mark and were similarly distributed between level I and level II hospitals. The total number of examined cases was stated as well as the missing values of individual variables per group. Level I and level II centers were compared with regard to ordinal or metric variables with the t-test and with regard to categorical variables with the Pearson’s chi-square-test. The primary endpoint of the analysis was the intra-hospital mortality rate. The mortality rate between level I and level II centers was adjusted by means of the RISC II score.

The standardized mortality ratio (SMR) is defined as quotient of the observed to the expected mortality–in our study, more precisely, lethality. If the calculated lethality rate (predicted by means of the RISC II score) is identical with the observed lethality, the SMR = 1. With an SMR < 1, the observed lethality rate is lower than the calculated one, more patients than expected survive. Conversely, an SMR > 1 indicates a higher lethality than expected.

The SMR was expressed with a 95% confidence interval based on a Poisson distribution for each level of health care. The significance levels of the unadjusted mortality rate and the SMR were precisely calculated by means of a two-sample t-test.

In a third step, a multivariable logistic regression model was generated that included the levels of health care (level I and level II), the RISC II score, as well as a corresponding interaction term (level of care x RISC II score) as an independent variable and hospital mortality rate as a dependent variable. In the multivariable logistic regression no missing values were included. Odds ratios (OR) and 95% confidence intervals were stated. The Receiver Operator Characteristic (ROC) curve was established as well as the Area Under the Curve (AUC) to test the regression model. The area under the curve (AUC), also called c-index or c-statistic, indicates how far a discrimination between alive and dead patients is possible. This can either be called “fit of the model” or “predictive power”. Values higher than 0.8 indicate a strong model for predicting patients outcome.

The levels of significance were set at 0.05 (two-sided). Statistical analyses were done with SPSS 23.0 for Windows (SPSS Statistics, IBM) and R 3.2.1 (The R Foundation for Statistical Computing).

## Results

### Expected versus treated patients in level I/level II centers

875 of 2596 patients documented in the study period were included in the study (Fig 2). 338 patients (39%) were treated in a level I center and 537 in a level II center (61%). Fig 1 shows the catchment areas of the individual level I and level II centers, Table 2 the comparison between the calculated and the observed patient numbers. The number of patients treated in level I centers was 148 higher than predicted. The total number of patients predicted to be treated in level II centers was not met. One level II center (hospital C in Table 2) showed the opposite result because 53 more patients were treated than predicted based on population density.

**Table 2:** Predicted and observed number of most severely injured patients (ISS ≥16).

	Center	Predicted	Observed	Difference (n/%)
**Level I centers**		**190 (22%)**	**338 (39%)**	**148 (+78%)**
	A	95	235	140 (+147%)
	B	95	103	8 (+8%)
**Level II centers**		**685 (78%)**	**537 (61%)**	**-148 (-22%)**
	C	86	139	53 (+62%)
	D	70	71	1 (+1%)
	E	45	37	-8 (-18%)
	F	121	89	-32 (-26%)
	G	110	77	-33 (-30%)
	H	80	50	-30 (-38%)
	I	50	26	-24 (-48%)
	J	123	48	-75 (-61%)

Predicted vs. observed number of patients. The predicted number of patients is calculated on the assumption that each patient was admitted to the closest possible level I or level II center by ground transportation.

### Demography

The mean age of most seriously injured patients (ISS ≥16) in the study region was 49 years; 73% were men and without any or only with minor pre-existing diseases (ASA I and II: 88%). Most patients had suffered blunt trauma (97%). These variables did not significantly differ between level I and level II centers (Table 3).

**Table 3:** Patient characteristics.

	Total	Level I	Level II	p-value
**Age** in years (n = 875)	48.7 (22.0)	46.9 (22.8)	49.8 (21.5)	0.071
**Sex** (n = 875)				
male	637 (73%)	242 (72%)	395 (74%)	0.526
female	238 (27%)	96 (28%)	142 (26%)	
**ASA** (n = 821)				
1 Healthy	501 (61%)	201 (61%)	300 (61%)	0.208
2 Mild systemic disease	219 (27%)	84 (25%)	135 (27%)	
3 Severe systemic disease	97 (12%)	45 (14%)	52 (11%)	
4 Life threatening disease	4 (<1%)	0 (0%)	4 (1%)	
Mean (SD)	1.52 (0.72)	1.53 (0.72)	1.51 (0.72)	0.753
*Missing values*	*54 (6%)*	*8 (2%)*	*46 (9%)*	
**GCS** preclinical (n = 838)	11.2 (4.7)	10.3 (4.8)	11.7 (4.6)	<0.001
*Missing values*	*37(4%)*	*8 (2%)*	*29 (5%)*	
**AIS head 4+**	296 (34%)	143 (42%)	153 (29%)	<0.001
**ISS** (n = 875)	27.4 (12.4)	30.9 (14.5)	25.2 (10.4)	<0.001
**NISS** (n = 875)	33.6 (14.5)	37.8 (16.7)	30.9 (12.3)	<0.001
**RISC** II (n = 875)	18.2 (28.4)	23.0 (31.7)	15.2 (25.7)	<0.001
**Type of trauma** (n = 853)				
Blunt force	825 (97%)	322 (96%)	503 (97%)	0.243
Penetrating	28 (3%)	14 (4%)	14 (3%)	
*Missing values*	*22 (3%)*	*2 (1%)*	*20 (4%)*	

Data show mean (SD) or number of patients (%, column percentage of all patients without missing values); GCS, Glasgow Coma Scale; ISS, Injury Severity Score; NISS, New Injury Severity Score; RTS, Revised Trauma Score; RISC II, Revised Injury Severity Classification Score II; p-value (comparison of Level I vs. Level II hospital level): t-test or Chi squared test.

However, level I and level II centers significantly differed in injury severity (GCS, ISS, and NISS) and the calculated mortality risk (RISC II). Level I centers admitted patients with more serious injuries and lower initial GCS (first GCS on scene before sedation/intubation), more patients with severe craniocerebral injuries, and patients with poorer survival prognosis (Table 3).

### Vital parameters and treatment parameters

Table 4 shows the vital parameters as well as the process and result parameters divided into preclinical situation, shock room, and further clinical course (secondary endpoints).

**Table 4:** Vital parameters and treatment parameters.

	Total	Level I	Level II	p-value
**Preclinical situation** (Table 4a)				
**Trauma resuscitation** (n = 859)				
Yes	45 (5%)	21 (6%)	24 (4%)	
No	830 (95%)	317 (94%)	513 (96%)	0.286
**Systolic blood pressure** (n = 786)				
RR ≤90	135 (17%)	69 (23%)	66 (14%)	0.001
Mean (SD)	122 (38)	118 (42)	125 (34)	0.015
*Missing values*	*89 (10%)*	*32 (9%)*	*57 (11%)*	
**Intubation** (n = 862)				
Yes	374 (43%)	192 (57%)	182 (35%)	
No	488 (57%)	143 (43%)	345 (66%)	<0.001
*Missing values*	*13 (1%)*	*3 (1%)*	*10 (2%)*	
**Cristalloid** (n = 756)	810 (543)	839 (597)	791 (506)	0.583
*Missing values*	*119 (14%)*	*46 (14%)*	*73 (14%)*	
**Colloid** (n = 572)	224 (359)	272 (408)	187 (311)	0.003
*Missing values*	*303 (35%)*	*86 (25%)*	*217 (40%)*	
**Time between emergency physician to emergency room** [min] mean (SD) (n = 494)	46.3 (22.6)	54.9 (22.2)	40.6 (21.0)	<0.001
*Missing values*	*381 (44%)*	*140 (41%)*	*241 (45%)*	
**Air rescue** (n = 855)				
Yes	375 (43%)	183 (55%)	192 (37%)	
No	480 (57%)	152 (45%)	328 (63%)	p<0.001
*Missing Values*	*20 (2%)*	*3 (1%)*	*17 (3%)*	
**Shock room** (Table 4b)				
**Systolic blood pressure** (n = 788)				
RR ≤90	116 (15%)	54 (19%)	62 (13%)	0.022
Mean (SD)	123 (35)	120 (36)	125 (34)	0.030
*Missing values*	*87 (10%)*	*46 (14%)*	*41 (8%)*	
**Base excess** (n = 699)	-3.1 (5.3)	-3.9 (5.9)	-2.5 (4.7)	0.026
*Missing values*	*176 (20%)*	*53 (16%)*	*123 (23%)*	
**Quick** (n = 829)	78.3 (22.3)	74.7 (23.4)	80.6 (21.2)	<0.001
*Missing values*	*46 (5%)*	*14 (4%)*	*32 (6%)*	
**Intubation** (n = 867)				
Yes	496 (57%)	237 (71%)	259 (49%)	
No	371 (43%)	99 (29%)	272 (51%)	<0.001
*Missing values*	*8 (1%)*	*2 (1%)*	*6 (1%)*	
**Transfusion of PRBC** (n = 875)				
0	781 (89%)	279 (83%)	502 (94%)	0.003
1–9	72 (8%)	42 (12%)	30 (6%)	
10+	22 (3%)	17 (5%)	5 (1%)	
Mean	0.9 (4.5)	1.6 (5.6)	0.5 (3.6)	0.002
**Transfusion of FFP** (n = 875)				
No	807 (92%)	289 (86%)	518 (96%)	<0.001
Yes	68 (8%)	46 (14%)	19 (4%)	
Mean (SD)	0.9 (4.4)	1.8 (5.8)	0.3 (3.0)	<0.001
**WBMS-CT or CCT** (n = 864)				
Yes	823 (95%)	321 (95%)	502 (95%)	0.998
No	41 (5%)	16 (5%)	25 (5%)	
*Missing values*	*11 (1%)*	*1 (<1%)*	*10 (2%)*	
**Time between shock room and CT** [min] (n = 811)	21.9 (14.8)	22.2 (13.0)	21.7 (15.8)	0.636
*Missing values*	*64*	*24*	*40*	
**Clinical course/Secondary endpoints** (Table 4c)				
**ICU** (n = 875)				
Yes	799 (91%)	322 (95%)	477 (89%)	
No	76 (9%)	16 (5%)	60 (11%)	0.001
**Intubation period** [days] (n = 496; all intubated patients, no missing values)	7.5 (9.0)	7.3 (8.2)	7.7 (9.6)	0.566
**Time on ICU** [days] (n = 799; all ICU patients, no missing values)	9.2 (10.5)	10.5 (10.6)	8.3 (10.3)	0.004
**Time in hospital** [days] (n = 875)				
Alive	19.0 (14.5)	20.3 (15.1)	18.3 (14.0)	0.073
Dead	4.4 (8.5)	4.7 (6.0)	4.2 (10.0)	0.687
Total	16.2 (14.7)	16.9 (15.1)	14.1 (15.7)	0.240
**Glasgow Outcome Scale** (GOS, n = 861)				
Death	170 (20%)	73 (22%)	97 (19%)	0.001
Persistent vegetative state	12 (1%)	9 (3%)	3 (<1%)	
Severe disability	63 (7%)	35 (10%)	28 (5%)	
Moderate disability	155 (18%)	50 (15%)	105 (20%)	
Low disability	461 (54%)	170 (50%)	291 (56%)	
Mean (SD)	3.8 (1.6)	3.7 (1.6)	3.9 (1.5)	0.028
**GOS summarized without deceased** (n = 691)				
Poor (persistent or severe)	75 (11%)	44 (17%)	31 (7%)	<0.001
Good (moderate or low)	616 (89%)	220 (83%)	396 (93%)	
*Missing values*	*14 (2%)*	*1 (<1%)*	*13 (2%)*	

Data show mean (SD) or number of patients (%, column percentage of all patients without missing value); Trauma resuscitation: Resuscitation with external mechanical chest compression; PRBC, Packed Red Blood Cells; FFP, Fresh Frozen Plasma; WBMS-CT, Whole Body Multislice Computed Tomography; CCT, Cranial Computed Tomography; ICU, Intensive Care Unit; p-value (comparison of level I vs. level II centers): t-test or Chi squared test.

Preclinically (Table 4A), level I and level II centers did neither differ with regard to the number of crystalloids (mean of 810 ml) administered nor regarding the percentage of preclinically reanimated patients (mean of 5%). Patients transferred to a level I center were more often shock patients (23% vs. 15%), more often required intubation (57% vs. 14%) and colloids, and were more often transported by air rescue (55% vs. 37%). The time between the arrival of the first emergency physician and hospital admission was longer for patients transferred to level I centers (55 vs. 41 min).

Preclinical time (time of accident till hospital admission) could not be evaluated in all cases due to incomplete documentation. We were able to evaluate 71% of all cases. Of these, the overall preclinical time was 63 min.

The difference between severe brain injury (42%) and preclinical intubation (57%) is based on the emergency physician system in Germany. Patients with GCS ≤ 8 should be intubated, as well as patients with multiple trauma and massive pain.

An additional evaluation of the transportation duration depending on the time of day showed a longer duration at night (darkness) compared to day (brightness), especially in case of air rescue and admission to level I centers (up to > 80 min.). This circumstance is due to a two-staged alert of the air rescue at night.

Process quality in the shock room (Table 4B) did not differ between level I and level II centers (surrogate variables: Proportion of computed tomogram (CT) diagnostics (95%) and time to CT scan 22 min). Of the 5% / n = 41 patients without primary CT diagnostics, n = 17 (41.5%) underwent emergency surgery, n = 16 (39.0%) died in the shock room and n = 8 (19.5%) received CT diagnosis later.

Level I and level II centers differed with regard to the clinical condition of patients: 19% of patients admitted to level I centers were in shock (13% in level II centers), had reduced base excess (-3.9 vs. -2.5), and poorer coagulation (Quick value 74.7 vs. 80.6). 71% of patients in level I centers were intubated (49% in level II centers). The proportion of blood products, particularly that of mass blood transfusions (PRBC ≥ 10), was higher in level I centers.

In the further course (Table 4C) of the treatment, more patients required monitoring in intensive care units (ICU) in level I centers than in level II centers (95% vs. 89%). Intubation times were similar between the two levels (mean of 7.5 days), but the length of stay in the ICU was longer in level I centers (10.5 vs. 8.3 days). However, the overall length of hospital stay did not differ between level I and level II centers, neither did the length of stay in the sub-groups survivors and non-survivors. Analysis by means of the GOS without consideration of the number of non-survivors showed very good or good outcome for 89% of patients in level I centers and for 93% of patients in level II centers (GOS 4 or 5; p<0.001).

### Univariable outcome analysis

The primary endpoint of the study was the mortality rate during the initial hospital stay. The total number of deaths was 170 (19.4%). Level I and level II centers did neither differ with regard to unadjusted, directly observed mortality rates (21.6% vs. 18.1%; p = 0.282) nor regarding adjusted mortality rates represented by the SMR (observed mortality rate/predicted morality rate calculated by means of the RISC II score) (0.94 vs. 1.18; p = 0.148; Table 5). Secondary endpoints such as intubation rate, intubation duration, length of stay, or GOS are depicted in Table 4S.

**Table 5:** Primary endpoint—Observed and predicted hospital mortality related to the hospital level.

	Total (n = 875)	Level I (n = 338)	Level II (n = 537)	p-value
Non-survivors	170	73	97	
RISC II predicted mortality (%)	18.2	23.0	15.2	
Observed mortality (% (95%-CI))	19.4 (16.4, 22.9)	21.6 (16.5, 27.9)	18.1 (14.4, 22.5)	0.282
Difference (observed/predicted) (%)	1.2	-1.4	2.9	
SMR (95%-CI)	1.07 (0.90, 1.26)	0.94 (0.72, 1.21)	1.18 (0.95, 1.48)	0.148

RISC II, Revised Injury Severity Classification II; SMR, Standardized Mortality Ratio (observed/predicted mortality)

### Multivariable outcome analysis

**Fig 3:**
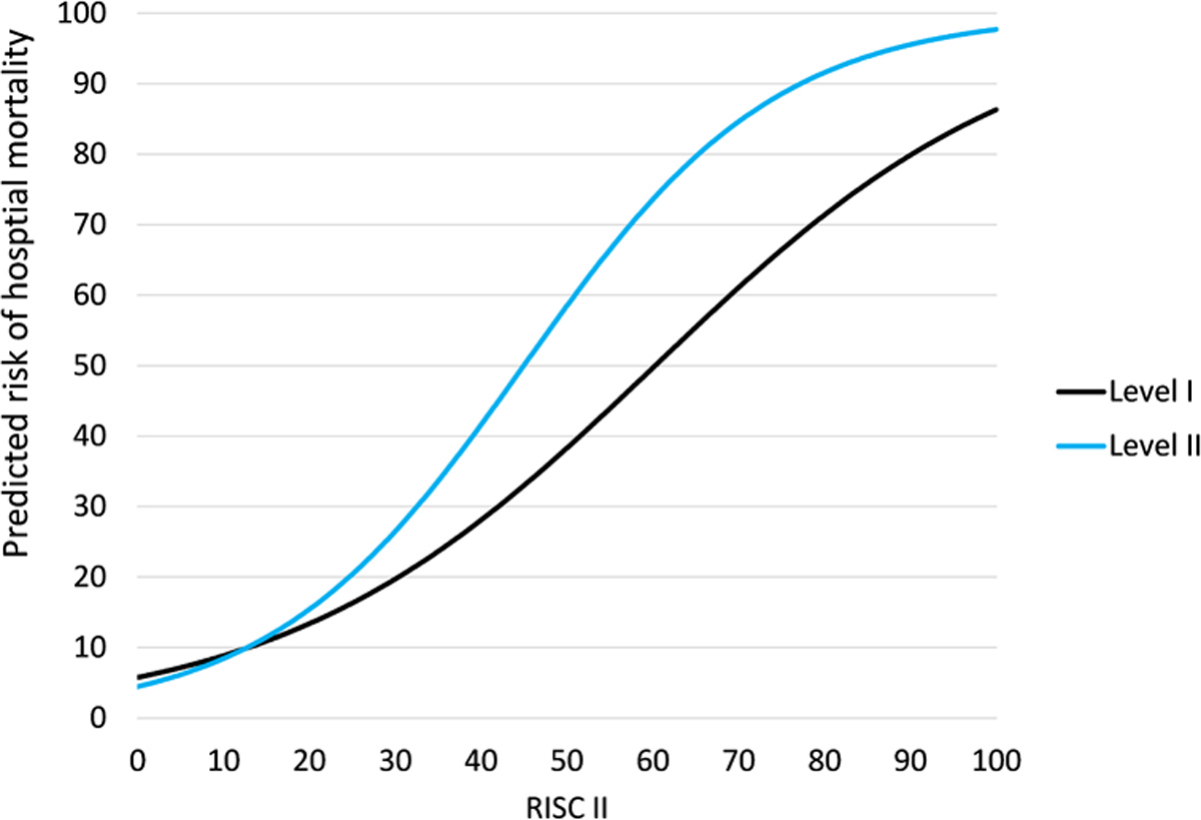
Risk of hospital mortality as a function of the RISC II score and hospital level according to the multivariable logistic regression model. Observed vs. predicted mortality rates by means of the regression model in level I and level II centers. The point of intersection in RISC II is 13%.

The multivariable logistic regression model showed a difference in the primary endpoint between level I and level II centers that depended on the level of the RISC II score, (significant interaction term with p = 0.007), see Table 6 and Fig 3.

**Table 6:** Multivariable logistic regression models on hospital mortality.

Predictor	OR (95%-CI)	p-value	AUC (95%-CI)
Level I (reference: level II)	1.31 (0.68, 2.51)	0.416	0.91 (0.88, 0.93)
RISC II	1.07 (1.06, 1.08)	<0.001	
Level I *RISC II	0.98 (0.96, 0.99)	0.007	

n = 875; RISC II, Revised Injury Severity Classification; OR, Odds Ratio; CI, Confidence-Interval

This figure shows that an increase in the RISC II score resulted in a higher increase in the survival probability in level I centers than in level II centers. In this study, the optimal cut-off of the logistic regression model-based probabilities for predicting death was 0.15; sensitivity was 79% and specificity 87%, which corresponds to an RISC II score of 23.0% in level I centers and of 19.5% in level II centers.

The two curves (level I vs. level II) intersect at an RISC II score of 13%. The probability of death did not differ between the centers of different health care levels when the probability was below 13%. However, above a probability of 13%, patients in level I centers had a significant survival advantage.

Cases below or above the point of intersection (RISC II ≤13% vs. >13%) show that 69% (n = 608) of cases are below and 31% (n = 267) above the point of intersection. 128 (48%) of the 267 patients with an RISC II score of >13% were treated in a level I center, resulting in a mortality rate of 48% (n = 61). 52% of patients with an RISC II score of >13% (n = 139) were treated in a level II center, yielding a mortality rate of 58% (n = 80).

For patients with an RISC II score of >13%, the number needed to treat is 10. Thus, in terms of figures, 10 patients with an RISC II score of >13% have to be treated in a level I center for 1 patient less to die.

Comparing the mean ISS of patients below and above the point of intersection of 13% in the RISC II score yields a difference of 23.1 (SD 7.3) vs. 37.3 (SD 15.7) (p<0.001). Because of the high range of dispersion, patients are only insufficiently separated by the ISS in this model. 32 patients with an ISS of <23.1 but an RISC II score of >13% were found but would have been undertriaged. In contrast, 35 patients had an ISS of >37.3 but an RISC II score of <13%.

**Fig 4:**
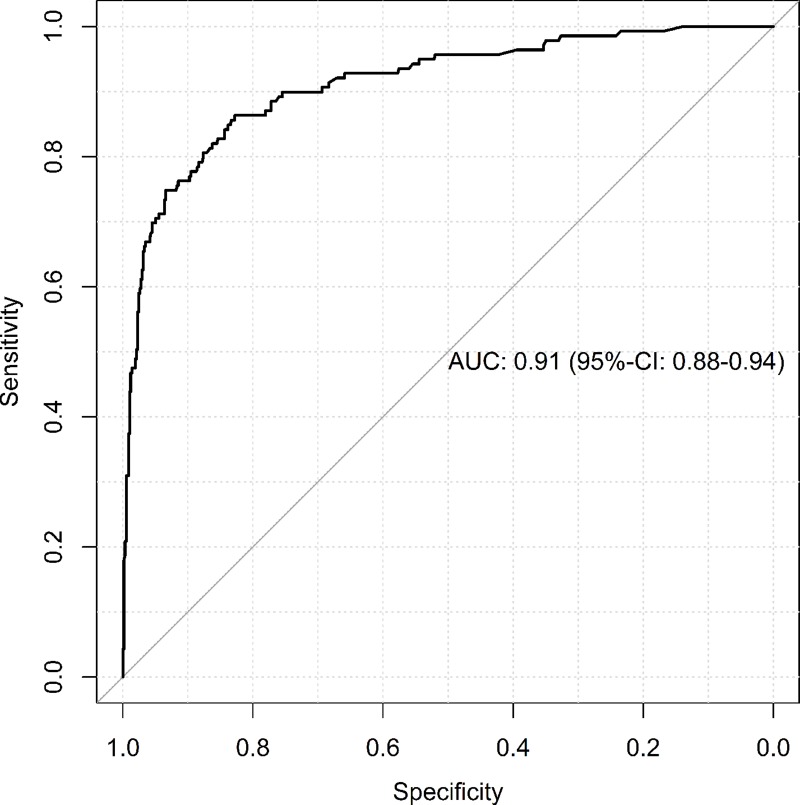
ROC analysis of predicted probabilities according to the multivariable logistic regression model on hospital mortality. Receiver operating characteristic (ROC) analysis of the logistic regression model. AUC, Area under the curve.

The predictive power of the logistic regression model was tested with the receiver operator characteristic (ROC) curve. The area under the curve (AUC) expressing the predictive power was 0.91 (Fig 4).

The ISS was also analyzed in an identical multivariate regression model. In this analysis, AUC at the optimal cut-off was 0.77, sensitivity 79%, and specificity 65%. Purely anatomical scoring systems such as ISS or the Abbreviated Injury Scale (AIS) have a lower level of discrimination and poorer predictive power of mortality than scoring systems in which anatomical and physiological parameters such as TRISS, RISC, and the RISC II score are taken into consideration [31]; therefore, no difference in ISS could be found between level I and level II centers in the case of increasing ISS values.

## Discussion

The key findings of the present study are:

1. The distribution of patients according to the levels of care showed a tendency to centralization in favor of level I centers. Overall, 61% of the most seriously injured patients with an ISS of ≥16 were treated in a level II center. If a geographically equal distribution of patients is assumed (ground transportation to the nearest trauma center), 78% of patients would have been treated in a level II center.
2. More seriously injured patients were more often treated in level I centers, which is the reason for the imbalance described above.
3. Univariate analyses did not yield any differences in outcome between the different care levels. Multivariate regression analysis shows a survival advantage of patients with higher calculated probability of death (over 13%) in level I centers.

To our knowledge, this is the first prospective study that compared health care of an entire trauma network–here that of the Trauma Network Eastern Bavaria–between level I and level II centers. Germany has a rather decentralized system / “over”-inclusive system of trauma care within trauma networks, particularly networks outside urban agglomerations. In the study region of 20,000 square kilometers with approximately 2.3 million inhabitants 2 level I centers and 8 level II centers with a total of 887 severely injured patients (ISS ≥16) were evaluated over 24 months. The distribution of these patients to such a large number of hospitals represents a different philosophy than e.g. the USA [32, 33]. However, Caputo et al. published in 2014 a systematic review including many North American studies [34]. The authors could not find a correlation of patient volume and mortality. Tepas et al. were also able to show that mortality increased with an excessive number of patients [35]. In contrast to this, other studies showed an improved survival rate correlated with high patient volume [36, 37]. Actually, the discussion of case numbers has not yet been finally resolved. For Germany, a survival advantage could be shown from a case-volume of 40 patients with an ISS ≥ 16 [38].

Taking every most seriously injured patient with an ISS of ≥16 to a level I center would place too much demand on the capacity of such centers. Therefore, it is both reasonable and necessary to also treat most seriously injured patients in level II centers to keep trauma care within the networks, particularly in rural areas.

No exact regulations or cut-offs of scoring system such as ISS exist for the choice of a target hospital, nor for air rescue use. However, during the day a primary air rescue is possible and is coordinated by the rescue directing center. At night two-staged alert by the ground-based emergency physician is mandatory. Brown et al. proposed indications for air rescue. Such an indication list is not yet implemented in the study region and could be helpful [39].

This decisions are made by the emergency physician on the scene and mainly based on the injury severity of the patient and the specialist departments available at the individual centers (for instance, patients with craniocerebral trauma should be taken to a center with a department of neurosurgery). The White Paper on the treatment of more seriously injured patients recommends a transportation time to a level I or level II center of less than 30 min [16, 17]. The POLYQUALY study has shown that 61% of patients with an ISS of ≥16 were treated in level II centers (537 of 865 patients). Demographical patient data (age, sex, and previous diseases) did not differ between level I and level II centers.

However, differences were found in injury severity and injury pattern. Patient selection by the emergency physicians on the scene may be the reason for the transfer of most seriously injured patients to level I centers. This assumption is supported by the comparison between the calculated and the observed number of patients. The overall number of most seriously injured patients treated in level II centers was higher than that in level I centers; however, the number of patients predicted to be treated in level II centers was 148 lower than calculated.

Not all multiple trauma patients (ISS≥16) have to be admitted to a level I center. Our study could not establish a limit value for the ISS that would have had an improved survival rate in level I centers. The RISC II score is unique—patients with a RISC II > 13% benefit from an admission to a level I center. It would be desirable to have a scoring system that allows a better distribution of trauma patients in the preclinical setting.

In consideration of the stipulated transportation time of less than 30 min, choosing a more distant level I center as a primary target hospital in a large area is only possible when air rescue is available. This study showed a high percentage rate of air rescues to level I centers (55%). According to Schweigkofler et al., the proportion of air rescues to level I centers in Germany is 42%, although most air rescue services in Germany only operate during daylight [40]. In the study region, a 24/7 dual use helicopter is also available during nighttime. Air rescue extends the preclinical period [40, 41], an effect that was also seen in the preclinical periods of level I centers in this study. This extension of time mainly seems to be caused by the necessary two-staged alarming of the air rescue by the ground based emergency physician on the scene and the further treatment by the Helicopter Medical System physician.

In the study region, primary air rescue operations are possible during the day; at nighttime, however, the air rescue needs to be requested by the emergency physician on the scene. This post-alarming further prolongs the preclinical time at night for the air rescue. Kleber et al. showed that there is no direct relationship between the preclinical period and the survival probability in the German emergency rescue system [41]. In 2004 and hence prior to the trauma network initiative of the German Society of Trauma Surgery, Biewener et al. reported a survival benefit of patients transferred to a level I center by helicopter [42]. Because the data sets of this study cannot answer the question when and why an emergency physician decides on a particular level I or level II center or when and why a ground-based emergency physician requests a helicopter, this topic should be the subject of further studies.

With regard to clinical care, it should be emphasized that level I and level II centers were comparable with regard to the proportion of patients receiving CT diagnostics as well as the period until the CT scan, which expresses the process quality of the present study. Ruchholtz et al. also reported a comparable percentage of CT scans but a longer period until the CT scan in level II centers [43].

The results of the vital parameters and treatment parameters emphasize that more seriously injured patients were more often transferred to level I centers. This result corresponds to the analysis by Ruchholtz [43]. The poorer outcome according to the Glasgow Outcome Scale (GOS) of the patients in our study mirrors the significantly higher proportion of patients with most severe craniocerebral trauma. The key question of the study was whether the survival probability was comparable between level I and level II centers. However, the patients treated in level I and level II centers significantly differed in injury severity as well as in the calculated probability of death (RISC II score).

Nevertheless, the univariate analyses–both unadjusted and adjusted–did not show any differences in the survival probability between the different hospital levels. Ruchholtz et al. described a similar result without any connection to the trauma network and the RISC I score [43].

The multivariate regression model showed a higher survival probability of patients in level I centers who–according to the RISC II score–had a probability of death of >13%. This result cannot be explained by the data obtained in this study. One reason may be the better equipment available in level I centers or advantages through better clinical routine measures due to the treatment of a higher number of patients. In our study, the mean number of patients with an ISS of ≥16 treated per year was 85 in level I centers and 34 in level II centers. Huber-Wagner et al. described a survival advantage of most seriously injured patients that depended on the number of patients treated per year, and this advantage already started to show after 40 patients [38]. The high volume over 200 ISS≥16, as required in the USA for level I hospitals, did not show any survival advantage in a systematic review [34].

In conclusion, the present study could show the effectiveness and high functionality of the rural Trauma Network of Eastern Bavaria: Overall, 875 most seriously injured patients with an ISS of ≥16 were treated in the 10 hospitals (2 level I centers and 8 level II centers) of the trauma network during the 24 months of the observation period. 61% of these 875 patients were treated in a level II center. Therefore, level II centers are indispensable for the treatment of patients in this region. Patients in level I centers had more serious injuries and a higher probability of death than the patients in the level II centers. For this reason, preclinical choice of the most appropriate hospital led to a certain centralization of most seriously injured patients. The different care levels were comparable with regard to the process quality. The quality of the results of the different care levels did not differ with regard to the mortality, neither in the unadjusted nor in the adjusted univariate analysis, despite the higher injury severity of patients in level I centers. Patients who had a probability of death of >13% (RISC II score) had a higher probability of survival in level I centers.

Trauma networks are being established in many countries in Europe with similar medical care structures, e.g. Austria, Switzerland or Spain. These results could be helpful in planning and implementing further trauma networks in other countries.

### Strengths and limitations of the study

The study included a large number of patients and was based on quality assured data. Yet, the completeness of cases in terms of collecting the data of all most seriously injured patients in the study region (full census) cannot be judged objectively. However, it may be assumed that the utilization percentage is close to the full census, because notification of patients is an essential criterion of qualification (minimum quantity regulation) for obtaining the trauma network certification.

The observed lethality may appear high compared to other studies with blunt trauma and ISS≥16. These studies use the injury mechanism and the ISS, however these parameters are not very useful for comparison as the ISS is an poor predictor for death for the TraumaRegister® database (area under the curve of receiver operating characteristic, AUC of ROC 0.82 (95% CI 0.81–0.83)) [31].

With a validated international mortality predictor scoring system could provide a true comparability. The TRISS shows an AUC of 0.91 (95% CI 0.91–0.92) for the study database [31]. On the other hand, the RISC II (AUC 0.95, 95% CI 0.95–0.96) is only validated for the TraumaRegister DGU^®^ [31]. The standardized mortality ratio (SMR), which would allow a comparability between different prediction scoring systems, is unfortunately not included in most previous studies.

A further problem may be the completeness of the investigated variables that was mainly over 90% in the univariate analyses. Despite the prospective two-tier manner of data acquisition, it was not always possible to collect all variables of a patient. However, there was no bias, because the missing values were equally distributed between the groups. With regard to the representativeness of the results, it should be noted that the study was conducted in the Trauma Network of Eastern Bavaria. Empirically, the conditions for the Trauma Network of Eastern Bavaria are similar to those for other networks in other regions and may thus be considered representative for trauma networks in Germany. In our opinion, the results of this small area analysis [44] can be transferred to other trauma networks in Germany. Minor differences may arise from fewer air rescue operations at night that may increase the status of level II centers in some other trauma network. The question if the results of this study can also be transferred to urban agglomerations with significantly more level I centers may be investigated in further studies.

## Conclusions

To our knowledge, this is the first study to investigate the effectiveness of a trauma network using the TraumaNetzwerk DGU^®^. This small area analysis [44] not only shows the effectiveness of trauma networks in rural areas in Germany but also that this concept results in broadly comparable outcomes at centers of the different levels of health care. The question if the results of this study can also be transferred to urban agglomerations in Germany and other trauma networks with similar philosophies in other countries may be investigated in future studies.

## Supporting information

S1 File Granted permission for Fig 1. Reprinted from http://www.d-maps.com/carte.php?num_car=6121&lang=de under a CC BY license, with permission from Daniel Dalet, original downloaded 2017.
(PDF)
