# Supplementary material for: A trauma network with centralized and local health care structures: Evaluating the effectiveness of the first certified Trauma Network of the German Society of Trauma Surgery
Source: PLoS One. 2018 Mar 14;13(3):e0194292. doi: 10.1371/journal.pone.0194292 (PMC5851627; doi:10.1371/journal.pone.0194292)
Supplement: S1 File — Reprinted from http://www.d-maps.com/carte.php?num_car=6121&lang=de under a CC BY license, with permission from Daniel Dalet, original downloaded 2017. (PDF) [file pone.0194292.s001.pdf]

http://d-maps.com's owner, I give to Dr. Antonio Ernstberger the permission to publish a figure built with a base map from my website, in an article for the journal PLOS ONE, under the Creative Commons Attribution License (CCAL) CC BY 4.0, in all formats i.e. print and digital.

Done in Digne-les-Bains (FRANCE), January 08, 2018.

Daniel DALET
